# Prevention of Alcohol Consumption Programs for Children and Youth: A Narrative and Critical Review of Recent Publications

**DOI:** 10.3389/fpsyg.2022.821867

**Published:** 2022-03-16

**Authors:** Rafael Sánchez-Puertas, Silvia Vaca-Gallegos, Carla López-Núñez, Pablo Ruisoto

**Affiliations:** ^1^Department of Health Sciences, Public University of Navarre, Pamplona, Spain; ^2^Department of Psychology, Particular Technical University of Loja, Loja, Ecuador; ^3^Department of Personality, Assessment and Psychological Treatments, School of Psychology, University of Seville, Seville, Spain

**Keywords:** alcohol, prevention, children, youth, review

## Abstract

**Background:**

Youth substance use is a public health problem globally, where alcohol is one of the drugs most consumed by children, and youth prevention is the best intervention for drug abuse.

**Objective:**

Review the latest evidence of alcohol use prevention programs in empirical research, oriented to all fields of action among children and youth.

**Methods:**

A narrative and critical review was carried out within international databases (PsychInfo, Pubmed, Web of Science, and Scopus) in August 2021 and was limited to empirical studies that appeared in the last five years (2017–2021). A flow diagram was used according to the PRISMA statements. Empirical research articles in English with RCTs and quasi-experimental design that included alcohol, children, and young people up to 19 years of age (universal, selective, or indicated programs) were included. The authors examined the results and conceptual frameworks of the Prevention programs by fields of action.

**Results:**

Twenty-two articles were found from four fields of action: school (16), family (2), community (2), and web-based (2), representing 16 alcohol prevention programs. School-based alcohol prevention programs are clinically relevant [*Theory of Planned Behavior*, *Refuse*, *Remove, Reasons, Preventure*, *The GOOD Life*, *Mantente REAL*, *Motivational Interviewing* (BIMI), *Primavera*, *Fresh Start*, *Bridges/Puentes*], they are effective in increasing attitudes and intentions toward alcohol prevention behavior, while decreasing social norms and acceptance of alcohol, reducing intoxication, and increasing perceptions with regards to the negative consequences of drinking.

**Discussion:**

This narrative and critical review provides an updated synthesis of the evidence for prevention programs in the school, family, community, and web-based fields of action, where a more significant number of programs exist that are applied within schools and for which would have greater clinical relevance. However, the prevention programs utilized in the other fields of action require further investigation.

## Introduction

Youth substance use represents a public health problem globally (Somani and Meghani, 2016; Stevens et al., 2020). The neurological development that occurs during childhood and adolescence combined with the onset of substance use (between the ages 15 and 19) (Blanco et al., 2018) becomes a particularly vulnerable stage that must be studied (Thorpe et al., 2020). Alcohol is one of the drugs most consumed by adolescents and young adults (Johnston et al., 2020). Particularly in the United States, 62.5% of underage alcohol users are binge alcohol users (Substance Abuse and Mental Health Services Administration [SAMHSA], 2018). Use and misuse of alcohol are associated with poor cognitive and executive functioning (Lees et al., 2020), increased risk of injury, death, and physical and sexual violence (Centers for Disease Control and Prevention [CDC], 2020), poor academic performance (Bugbee et al., 2019; Chai et al., 2020), and increased exposure to social risks and early sexual activity (Boisvert et al., 2017). Moreover, young people who drink alcoholic beverages are more likely to use tobacco and other drugs and develop risky sexual behaviors (Lee et al., 2018).

Currently, alcohol abuse is characterized by high relapse rates, around 70–80% within a year (Dousset et al., 2020). In 2017, a systematic review found that children are aware of and able to recognize alcohol and its effects, suggesting the importance of starting prevention as soon as possible (Jones and Gordon, 2017). For this reason, the National Institute on Drug Abuse (National Institute on Drug Abuse [NIDA], 2020a) considers prevention the most cost-effective intervention for drug abuse. Unfortunately, there is no single accepted concept to define “drug use prevention”.

The European Monitoring Center for Drugs and Drug Addiction (European Monitoring Centre for Drugs and Drug Addiction [EMCDDA], 2015) defines “prevention” as any policy, program, or activity to (at least partially) delay or, directly or indirectly reduce drug use, including the possibility of minimizing drug use, limiting the negative consequences for health and social development or the progression of problematic drug use. As well it states that preventive actions among young people should be initiated early in their lives (European Monitoring Centre for Drugs and Drug Addiction [EMCDDA], 2021). In addition, substance use prevention also emphasizes protection against the initiation, progression, and maintenance of drug use, training in healthier coping strategies and social skills, or the development of social policies that reduce the availability and accessibility (such as prices) of alcohol (Becoña, 2007; Caywood et al., 2015). Overall, evidence-based prevention programs are encouraged (Harrop and Catalano, 2016; Funk et al., 2020).

### Drug Use Prevention Programs

Most prevention programs seek to reduce the number and type of drugs consumed, delay the age of onset of drug use, eradicate the impact of negative consequences among those who already use drugs or have abuse/dependence problems, as well as reduce risk factors and enhance protective factors by providing healthy alternatives to consumption (Becoña and Cortés, 2011; National Institute on Drug Abuse [NIDA], 2020b). Most programs are based on three essential components (Reno et al., 2000; Tobler et al., 2000): reducing supply (reducing access and availability of drugs), reducing or delaying drug demand, and limiting health and social consequences.

Prevention, conceptualized as an intervention that occurs before the onset of the disorder, is usually classified into three types: universal, selective, or indicated (Griffin and Botvin, 2010). Universal prevention programs are aimed at the general population. These are less intense and expensive than the other two types (for example, this would include school-level preventive activities that promote skills to refuse drug offers, improve self-esteem, and other factors that protect against substance abuse) (Espada et al., 2003; Griffin and Botvin, 2010). Selective prevention programs are aimed at high-risk groups within the general population and indicated prevention strategies are aimed at a specific subgroup of the community, which are usually consumers who show premature signs of danger for the development of addictive disorders (Griffin and Botvin, 2010; Becoña and Cortés, 2011). That is, indicated prevention targets those who already show early signs of substance use problems, engage in substance abuse, or other high-risk behaviors associated with drug consumption (Griffin and Botvin, 2010).

In addition, prevention programs can be developed in different fields of action, such as family-based, that encourage positive interaction between parents and children in connection with different developmental milestones (Van Ryzin et al., 2016); school-based, that provide a safe space for children and adolescents to discuss their problems with their friends and peers, and allow for regular supervision, as children spend a significant amount of time each day at school (Spanemberg et al., 2020); community-based, that refers to the community’s efforts to prevent consumption by its members (Hafford-Letchfield et al., 2020); and recently, mindfulness-based intervention (MBI), that includes paying attention in the present moment in a particular way: on purpose and without judgment (Korecki et al., 2020).

Recent systematic reviews of prevention programs have focused solely on either family-based (Van Ryzin et al., 2016; Ballester et al., 2020), school-based (Tremblay et al., 2020), or community-based fields of action (Melendez-Torres et al., 2016; Hafford-Letchfield et al., 2020). However, most programs are included within other broader programs whose objective is to improve the school climate and prevent bullying (Spanemberg et al., 2020) or are specific micro-interventions, such as interventions based on mindfulness (Korecki et al., 2020). This study aims to critically review the latest empirical evidence of alcohol prevention programs in children and youth.

## Materials and Methods

A narrative and critical review was carried out in international databases (PsychInfo, Pubmed, Web of Science, and Scopus) in August 2021 and was limited to empirical studies that appeared in the last five years (2017–2021). The keywords used were: “alcohol”, “child*”, “young adults”, and “prevent*”. The Boolean connector used was AND.

**FIGURE 1:**
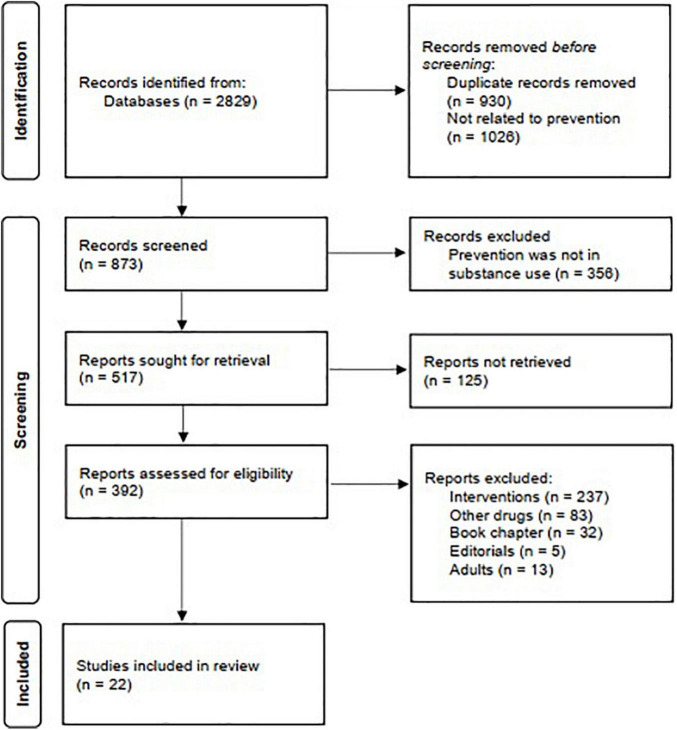
Flow diagram of search and selection of articles according to PRISMA statements.

The criteria to carry out the selection process were the following: empirical research articles with randomized controlled trials (RCTs) and quasi-experimental design that included alcohol as a variable, that the target group constituted children and young people up to 19 years of age (universal, selective or indicated programs), and that the studies had been published in English journals of high quality and impact factor. Although this is not a systematic review, a flow chart according to the PRISMA statements (Moher et al., 2009; Page et al., 2021) was used for this narrative and critical review (Figure 1). The records were removed before screening in the identification stage because they were duplicates or unrelated to the intervention. In contrast, the papers were eliminated in the first stage of the screening (records screened) because the prevention was not in substance use.

The authors examined the results and conceptual frameworks of the prevention programs by fields of action in children and young people up to 19 years: Do these interventions reduce the amount and/or frequency of use? Does the intervention influence other variables such as attitudes, intentions, perceptions, or social norms about alcohol consumption? The evidence reviewed along with the conceptual frameworks and key results of the reviewed articles are described in Supplementary Table 1.

## Results

### Characteristics of Included Studies

Of the 22 included studies, 16 were randomized controlled trials (72.73%), and 6 (27.27%) were quasi-experimental (see Supplementary Table 1).

### Description of the Programs

Supplementary Table 1 summarizes basic information of the 16 prevention programs reviewed, the intervention, the conceptual framework, and their results. The school prevention programs found were: the *Triad*; *Primavera*; *Bridges*/*Puentes*; *Mantente REAL*; *Preventure*; *Refuse, Remove, Reasons* program (*RRR*); *Fresh Start*; based in *Motivational Interviewing* program (*BIMI*), *Unplugged* (*Tamojunto*); *The GOOD Life*; pragmatic prevention, and a program based in Theory of Planned Behavior. The family prevention programs found were *Media Detective Family* and *Effekt*. The community prevention programs found were *Öckerö Method* and a program based on the Theory of Planned Behavior. Finally, the web-based prevention program was *RealTeen*.

Almost all reviewed alcohol prevention programs were universal; that is, they intervened before the initiation stage, except one (Lammers et al., 2017), which was a selective prevention program. The fields of action ranged from school (16 studies, 72.7%), family (2 studies, 9.1%), community (2 studies, 9.1%), to web-based (2 studies, 9.1%) prevention programs. Some of these programs were aimed at preventing the use of other drugs in addition to alcohol. All studies explicitly explained subject randomization and pooling in their analyses, mainly involving subjects, groups, or clusters (classes or schools). The studies showed heterogeneous sample sizes, ranging from *N* = 45 to 6,658; and *n* = 23 to 3,340 participants in the experimental group. Two studies (Schwinn et al., 2017; Park et al., 2021) applied their programs exclusively to girls, while the remaining investigations were developed for both boys and girls. The age of the children and youth ranged from 10 to 19 years old. Outcomes ranged from immediately post prevention to 5-year assessment period follow-ups.

### Prevention Programs by Fields of Action

#### Conceptual Framework of School-Based Prevention Programs

All the programs were universal programs (except Lammers et al., 2017, who studied adolescents with previous drinking experience) applied to students in a longitudinal design, regardless of their risk of alcohol consumption. The programs focused on social skills, intention and motivation, personality traits, and risk and protective factors for alcohol use. Considering the stage of development, children and young people begin to consume alcohol due to social and psychological pressure from peers, family, culture, and the media, since they lack or do not yet have all the skills and knowledge to recognize and resist such pressure. In other words, the programs seek to avoid alcohol consumption by resisting external pressure and increasing coping skills, considering their personality traits, and also by allowing children and young people to analyze their negative emotional reactions, irrational thoughts and behavioral intention while maintaining a negative attitude toward alcohol consumption to promote healthy behavior.

Several programs seek to develop social skills to reduce the effects of the social influence of alcohol consumption. Sanchez et al. (2017, 2018, 2019); Valente et al. (2019), and Vigna-Taglianti et al. (2021) applied the *Unplugged* program, based on the social influence model, supporting the development of general social skills that are thought to reduce the effects of social influence by modifying attitudes, beliefs, and normative perception (Kreeft et al., 2009). *The GOOD Life* program [applied by Vallentin-Holbech et al. (2019)] is based on the social norms approach and aims to change the overestimation of peer use. The *Refuse, Remove, Reasons* (Mogro-Wilson et al., 2017) program (RRR) uses social learning theory and the mutual aid model that combines social resistance skills training and normative education. Beckman et al. (2017) focused on one of the three components of the *Triad* program, namely, the alcohol use prevention component (called *Fighting Drugs*). *Primavera* (Diaz et al., 2021) uses health promotion as a reference basis (Dudley et al., 2015) and is mainly based on experiential learning (Potvin and Jones, 2011) via the development of psychosocial skills for preventing adolescent alcohol and tobacco use.

Among the programs that are based on behavioral intention are Kim et al. (2021) (web-based) and Onrust et al. (2017) (*Fresh Start* program), based on the *Theory of Planned Behaviour* which states that behavioral intention is the direct determinant of changing to healthy behavior and that people with solid intentions strive to achieve the goal of not drinking and are more easily motivated to change their behavior (Ajzen and Madden, 1986). *Mantente REAL* (Kulis et al., 2020) (uses ecological risk and *Resiliency Theory*, *Communication Competence Theory*, and *Narrative Theory*), a Spanish language version of *keepin’ it REAL* (kiREAL), increases the use of culturally accepted drug resistance skills and promotes non-permissive norms and attitudes about substance use (Gosin et al., 2003). *Motivational Interviewing* (BIMI) (Reyes-Rodríguez et al., 2019) seeks to identify a present or latent problem about consumption and from there motivate the person to carry out a change (Pilowsky and Wu, 2013).

*Bridges/Puentes* (Gonzales et al., 2018) emphasizes risk reduction (prevention) as well as positive youth development (promotion) in multiple domains (family, school, and peers) (Koning et al., 2013); Hodder et al. (2017a) used a *pragmatic intervention* to implement available programs and resources targeting individual and environmental ‘resilience’ protective factors.

Finally, *Preventure* is a selective prevention program based on *Cognitive Behavioural Therapy* with a personality-targeted approach (Lammers et al., 2015).

#### Outcomes of School-Based Prevention Programs

Sanchez et al. (2017) found that the *Unplugged* program (culturally adapted to Brazil) seemed to increase alcohol use initiation (9 months follow-up). Three studies based their results on the intervention performed by Sanchez et al. (2017, 2018) did a 21-month follow-up and found an increase in alcohol use in intervention and control groups. Sanchez et al. (2019) showed that the program’s effect on drug use via normative beliefs was not statistically significant. Valente et al. (2019) found that the impact of the intervention is unlikely to be conditioned to parenting style dimensions. Moreover, Vigna-Taglianti et al. (2021) applied *Unplugged* in Nigeria (culturally adapted) and found that the program significantly reduced the prevalence of recent alcohol use; furthermore, the program prevented regress across stages of alcohol use.

Several programs made it possible to reduce alcohol consumption. Diaz et al. (2021) used the *Primavera* prevention program and showed that children from the control group were less likely to report current alcohol use at the end of the first year of the intervention. Gonzales et al. (2018) used the *Bridges/Puentes* program, which significantly reduced the likelihood of developing an alcohol use disorder five years later. The results of Kulis et al. (2020) (*Mantente REAL* prevention program) showed relatively less frequent use of alcohol, and higher risk students reported relative reductions in the frequency of alcohol use, especially males. Mogro-Wilson et al. (2017), using the RRR, found significantly reduced inebrity from alcohol use, decreased social norms and acceptance of alcohol, and increased perceptions about negative perceptions and consequences of alcohol use. A brief intervention (based on *Motivational Interviewing*) was applied by Reyes-Rodríguez et al. (2019), showing a significant reduction of risk levels of alcohol consumption six months later.

Three investigations found no positive effect of the interventions. The *Triad* prevention program was applied by Beckman et al. (2017), who did not see an impact on the likelihood of drinking alcohol or drinking to intoxication. Hodder et al. (2017a) used a *pragmatic intervention*. There was no difference in the prevalence of any measure of substance use between intervention and control students, nor was there any difference for an aggregate or individual measure of personal and environmental protective factors. Vallentin-Holbech et al. (2019) applied to *The GOOD Life* program. The outcome shows that the intervention effect was insignificant for the frequency of binge drinking, and with regards to overestimated peer drinking, higher preventive effect sizes were observed for higher levels of exposure, satisfaction, and recall.

Finally, Park et al. (2021) (*Theory of Planned Behavior*) applied the program to girls. They found improved alcohol-related knowledge and converted individuals’ positive expectations of alcohol to negative ones. On the other hand, Kim et al. (2021) found significant improvements in attitudes and intention toward alcohol drinking prevention behavior. The results observed by Onrust et al. (2017) (*Fresh Start*) were minimal but significant effects on attitudes toward alcohol were seen. The *Preventure* program (Lammers et al., 2017) found significant intervention effects on reducing alcohol use within the anxiety sensitivity group and reducing binge drinking and binge drinking frequency within the sensation-seeking group.

#### Conceptual Framework of Family-Based Prevention Programs

Two universal family-based prevention programs (Scull et al., 2017; Tael-Öeren et al., 2019) focused on parent-child dyads. They seek the development of parental control skills, parenting behaviors, and the establishment of clear limits or rules, as well as their children’s peer and social resilience skills, and maintaining parental restrictive attitudes toward adolescents’ alcohol use over time.

Tael-Öeren et al. (2019) applied *Effekt* (previously known as the *Örebro Prevention Program*) sought to delay and reduce adolescents’ alcohol use by maintaining parental restrictive attitudes toward adolescents’ alcohol use over time (Koutakis et al., 2008). The *Media Detective Family* was an online media literacy education substance abuse prevention program that parents and their children complete together, whose goals are to enhance the message interpretation process skills of both parents and children and reduce children’s use of alcohol and tobacco (Scull et al., 2017).

### Outcomes of Family-Based Prevention Programs

The *Effekt* prevention program (Tael-Öeren et al., 2019) positively affected parental attitudes, but it failed to delay or reduce adolescents’ alcohol consumption. The *Media Detective Family* prevention program, applied by Scull et al. (2017), found that children reported a significant reduction in their use of substances over time.

#### Conceptual Framework of Community-Based Prevention Programs

Two universal community-based prevention programs (Park et al., 2021; Svensson et al., 2021) focused on strengthening the community as a more protective environment from alcohol use for children and youth. They provided information and offered education about alcohol and its associated risks, reduced access to alcohol, promoted health, improved advocacy for the media, strengthened restrictions, attitudes, and approaches to youth alcohol use among parents, other adults, and the community.

The study carried out by Park et al. (2021) used the *Theory of Planned Behavior* explained above. *Öckerö Method* was a program whose goal was delaying the onset of alcohol use and reducing alcohol consumption among youths by strengthening restrictive attitudes and approaches to youth alcohol consumption among parents and other adults (Svensson et al., 2021).

#### Outcomes of Community-Based Prevention Programs

The results of both studies were heterogeneous. Svensson et al. (2021) (*Öckerö Method*) did not show empirical evidence that the intervention affected adolescents’ drinking habits or their perceptions of their parents’ attitudes toward adolescent drinking. On the other hand, Park et al. (2021) improved alcohol-related knowledge and converted individuals’ positive expectations of alcohol to negative ones.

#### Conceptual Framework of Web-Based Prevention Programs

Although some programs from different fields of action use the web as a tool (online), two studies have been found that do not fit into any of these fields and are described simply as web-based and gender-specific interventions (girls). *RealTeen* prevention program [used by Schwinn et al. (2017) and Schwinn et al. (2019)] is based on *Social Learning Theory*. It is aimed at helping girls navigate the risks associated with peer and social influences to use alcohol. This intervention focuses on goal setting, decision making, puberty, body image, coping, drug knowledge, and refusal skills.

#### Outcomes of Web-Based Prevention Programs

Schwinn et al. (2017) found that girls reported less binge drinking, higher alcohol refusal skills, coping skills, and lower peer drug use rates at one-year follow-up. On the other hand, Schwinn et al. (2019) [based on data from Schwinn et al. (2017)] didn’t find reductions in binge drinking at 2-and 3-years follow-up.

## Discussion

In this research, the latest evidence of alcohol use prevention programs in empirical research oriented to all fields of action in children and youth has been reviewed, utilizing data from the last five years (2017–2021). Programs aimed at children and young people were reviewed due to the importance of prevention in these stages of development. Twenty-two studies were identified representing 16 prevention programs. The fields of action ranged from school (16 studies), community (2 studies), family (2 studies) to web-based (2 studies) prevention programs. Despite the significant heterogeneity of programs (both in sample size and follow-ups) and the difference in the number of studies for each field of action, most prevention programs are clinically relevant, given their results. The effects of universal prevention programs are generally miminal (Onrust et al., 2016), and may be attributed to the inconsistency of program content and the diversity of the theoretical frameworks, as well as the boomerang effect (whereby trying to correct exaggerated perceptions of overall prevalence, consumption increases rather than protects against alcohol consumption (Hopfer et al., 2010)).

### School-Based Prevention Programs

Beginning with school-based prevention programs based in *Theory of Planned Behavior* (Kim et al., 2021), *Refuse, Remove, Reasons* (Mogro-Wilson et al., 2017), *Preventure* (Lammers et al., 2017), *The GOOD Life* (Vallentin-Holbech et al., 2019), *Mantente REAL* (Kulis et al., 2020), *Motivational Interviewing* (Reyes-Rodríguez et al., 2019), *Primavera* (Diaz et al., 2021), *Fresh Start* (Onrust et al., 2017), and *Bridges/Puentes* (Gonzales et al., 2018), all are effective in increasing attitudes and intention toward alcohol prevention behavior, decreasing social norms and acceptance of alcohol, reducing insobriety, and increasing perceptions about negative consequences of drinking. In contrast to this, the prevention program called *Unplugged* not only did not show effectiveness in the study by Valente et al. (2019), but even seemed to increase alcohol use initiation in the studies by Sanchez et al. (2017) and Sanchez et al. (2018). However, it was effective in Nigeria (Vigna-Taglianti et al., 2021). The “*pragmatic prevention*” (Hodder et al., 2017a) was not effective either, possibly because the school staff selected the type, manner, and order of implementation of curriculum resources and programs; such interventions are less likely to be effective than non-pragmatic approaches (Yoong et al., 2014). *The Triad* (Beckman et al., 2017) did not affect the likelihood of drinking alcohol, applying only one of the program’s three components.

Other systematic reviews and meta-analyses have found similar results on school-based prevention programs. For example, the systematic review by Tremblay et al. (2020) found that 70% of the programs demonstrated reductions in the use of substances, including both alcohol and drugs; and the systematic review and meta-analysis by Melendez-Torres et al. (2018) concludes that this type of intervention was broadly effective for reducing specific alcohol and drug use. However, opposite results have also been found. The systematic review conducted by Hodder et al. (2017b) found that the universal school-based interventions that address adolescent ‘resilience’ protective factors as part of any intervention approach are ineffective for reducing adolescent alcohol use.

The school-based prevention programs that are most likely to be successful are those that combine the practice of social skills and the transmission of educational knowledge (Tobler et al., 2000; Botvin and Griffin, 2007) but also those programs that target their interventions at more than one risk factor (Griffin and Botvin, 2010; Hale et al., 2014). Among the components that increase the effectiveness of the programs are: the strengthening of social, emotional, behavioral, cognitive, and moral competencies; the increase in self-efficacy; improving social relationships with adults, peers, and younger children; and longer interventions (Catalano et al., 2004; Cairns et al., 2014). However, research is lacking in universal alcohol prevention programs with primary and lower grade students that promote personal and social life skills (Onrust et al., 2016), including self-control, promotion of self-esteem, and problem-solving skills (Onrust et al., 2016), supplemented with the offer of healthy alternatives, work with parents and peer education (MacArthur et al., 2016; Onrust et al., 2016).

Kim et al. (2021); Onrust et al. (2017) (*Fresh Start*), and Mogro-Wilson et al. (2017) (*RRR*) found improvements in attitudes and intention toward alcohol consumption, decreased social norms and acceptance of alcohol, and increased perceptions about negative consequences of alcohol use. According to a systematic review (Jones and Gordon, 2017), children’s attitudes toward alcohol become more positive as they get older. For this reason, early interventions must be applied to delay or prevent the formation of positive attitudes, perceptions, and social norms toward alcohol and follow alcohol consumption prevention guidelines that allow students to control the pressures of alcohol consumption (Kim et al., 2021), delaying consumption.

Among the programs that target their intervention at more risk factors is *Unplugged*, which supports the development of life skills (communication, assertiveness, critical thinking, coping strategies, goal setting, decision making, and problem-solving) and skills to resist the pressure to use drugs (Kreeft et al., 2009). The program seeks to strengthen adolescents’ personal and interpersonal skills that reduce the effects of social influence by modifying attitudes, beliefs, and normative perceptions (Sussman et al., 2004; Giannotta et al., 2014). The change in drinking behavior, which did not decrease but rather increased after nine months (Sanchez et al., 2017) and at the 21-month follow-up (Sanchez et al., 2017) in Brazil, could be due to the context and probably influenced by many factors, such as the age of the pupils, prevalence of use, social pressure, and, not last, fidelity of implementation. In addition, adaptations have to ensure that the intervention content, language, examples, and delivery methods are culturally appropriate, relevant, and acceptable to the new population (Castro et al., 2004).

Some research has found that the effectiveness of preventive interventions in schools may depend on implementation parameters such as acceptance of the building blocks, the scope of intervention, and mode of delivery (Cuijpers, 2002; Perkins and Craig, 2006; Domitrovich et al., 2008). In other words, the students’ attention would increase if the intervention is attractive to them, facilitating their ability to retain the central messages (Domitrovich et al., 2008; Durlak and DuPre, 2008); Vallentin-Holbech et al. (2019) (*The GOOD Life*) studied these variables, finding that no significant effects for any level of exposure were found, neither for satisfaction, nor recall for binge drinking. Further research is required to determine the impact of these variables on other prevention programs.

Students with anxiety sensitive traits have shown higher levels of alcohol use and drinking problems in previous research (Sher et al., 2000; Krank et al., 2011), and Lammers et al. (2017) (*Preventure*) found significant intervention effects on reducing alcohol use within the anxiety sensitivity group, reducing binge drinking and binge drinking frequency. This is one of four personality profiles at higher risk of developing alcohol problems (sensation seeking, impulsivity, anxiety sensitivity, and negative thinking) (Comeau et al., 2001).

The application design of the programs must be taken into account. Although most were randomized controlled trials, three were quasi-experimental (Beckman et al., 2017; Mogro-Wilson et al., 2017; Kim et al., 2021). A limitation of the quasi-experimental studies is that the program’s identification of a causal effect is based on the assumption that the intervention and control schools would have had the same trend in alcohol consumption without the intervention, which is impossible to test.

### Family, Community, and Web-Based Prevention Programs

Similarly, prevention programs based on the family and the community do not allow conclusions to be reached on their effectiveness, since only Scull et al. (2017) (family-based) found a reduction in alcohol consumption among children. Park et al. (2021) (community-based) found that the program improved alcohol-related knowledge and converted individuals’ positive expectations of alcohol to negative ones.

Two systematic reviews (Allen et al., 2016; Kuntsche and Kuntsche, 2016) and a meta-analysis (Van Ryzin et al., 2016) analyzed the effectiveness of family-oriented alcohol prevention offerings, allowing for the conclusion that these programs may have preventive effects on alcohol consumption in young people. For the most part, they aimed to strengthen parental behavior and self-efficacy to improve alcohol-related family communication. Both parents and youth worked on their life skills and leisure activities in family programs. Van Ryzin et al. (2016) found that the overall impact across different programs was small to moderate.

Moreover, two systematic reviews of community programs of mentoring to prevent or reduce alcohol found a significant overall effect on alcohol consumption (Thomas et al., 2013; Tolan et al., 2014); Toomey and Lenk (2011) found that programs that change the community environment can reduce alcohol use and related problems among youth. Strategies that lead to a general increase in the price of alcoholic products, increased regulation, control, and penalties for providing alcohol to minors, and restricting alcohol advertising could be recommended (Paschall et al., 2009).

On the other hand, two web-based prevention programs (Schwinn et al., 2017, 2019) applied to girls showed, in the same way, their clinical importance as gender-specific prevention, since they reported less binge drinking and higher alcohol avoidance skills and coping skills, even at 1-year follow-up. From these only two results, no general conclusions can be reached, apart from the fact that it is a gender-specific prevention program; however, a web-based prevention program applied to first-year college students showed a reduction in alcohol consumption (Gilbertson et al., 2017), so more research is required on this type of program.

The results obtained by Tael-Öeren et al. (2019) using *Effekt* are possibly due to it being an adaptation aimed at 11-year-old children, while additional versions were designed for 13-year-old children (Koutakis et al., 2008), which resulted in the choice of different measures to address the initiation of alcohol consumption. Beckman et al. (2017) applied only the intervention “Fight against drugs” of *The Triad*, not the other interventions associated with other behavioral issues. It may be that using all the themes is more effective, as the entire program addresses various risk behaviors.

### Limitations

Among the limitations of this research are studying alcohol consumption in populations that include young age groups which still do not drink or are starting to do so, so the evaluation and the results should be analyzed with caution. Furthermore, this is not a systematic review, which is restricted to the findings of the last five years. Knowing the most current evidence of prevention programs in children and youth in the different fields of action implies comparing varying program interventions, conceptual frameworks, and results, which limits the generalization of results and conclusions.

## Conclusion

Individual studies are certainly not sufficient to conclude for or against the large-scale implementation of, for example, family, community, or web-based alcohol prevention programs in the clinical setting. In light of how alcohol use can be countered in the population, prevention science can support practice and policy by providing reliable knowledge for children, adolescents, and youth-oriented addiction prevention. Research and clinical practice must be evidence-based. Its implementation must take into consideration accumulated practical knowledge and the particularities of the target group and the local context. In only this way can a consensus be reached on the methods by which causality of the connection between alcohol-related issues and consumer behavior be established.

Future research should continue to seek evidence of the most effective programs but also expand into new, under-studied fields, such as technology-based substance use prevention programs (Stinson et al., 2020) and mindfulness-based programs (MBP), which should be systematically tested in this population (Riggs and Greenberg, 2019). In addition, studies are needed to assess the quality of investigations and reviews that employ prevention programs to reach more effective conclusions (Shea et al., 2017), such as standardizing follow-ups.

Given the individual and social costs of alcohol use in youth, and increasingly in children, as a public health problem, it is the responsibility of the family, the school, the community, and the state to know the most current evidence of alcohol prevention programs. To this end, this narrative and critical review provides an updated synthesis of the evidence for prevention programs in the school, family, community, and web-based fields of action, where a greater number of programs applied in the school which ultimately carry greater clinical relevance. However, the prevention programs used in the other fields of action require further investigation.

## Author Contributions

PR, CL-Z, and RS-P: record review, evaluation of full-text studies for inclusion, and data extraction. RS-P: writing—original draft preparation. PR, CL-Z, and SV-G: writing—review and editing the final version. All authors have read and agreed to the published version of the manuscript.

## Conflict of Interest

The authors declare that the research was conducted in the absence of any commercial or financial relationships that could be construed as a potential conflict of interest.

## Publisher’s Note

All claims expressed in this article are solely those of the authors and do not necessarily represent those of their affiliated organizations, or those of the publisher, the editors and the reviewers. Any product that may be evaluated in this article, or claim that may be made by its manufacturer, is not guaranteed or endorsed by the publisher.

## Supplementary Material

Supplementary Table 1 Studies of alcohol use prevention programs for children and youth.
