# Supplementary material for: Prevention of Alcohol Consumption Programs for Children and Youth: A Narrative and Critical Review of Recent Publications
Source: Front Psychol. 2022 Mar 16;13:821867. doi: 10.3389/fpsyg.2022.821867 (PMC8965835; doi:10.3389/fpsyg.2022.821867)
Supplement: Supplementary Table 1 — Studies of alcohol use prevention programs for children and youth. [file Table_1.pdf]

Table 1. Studies of alcohol use prevention programs for children and youth

N	Program / Studies / Country	Field of action	Sample* (prevention (%girls)/ control (%girls)), age	Design	Intervention	Conceptual framework	Results
	The Triad / Beckman et al. (2017). / Sweden	School	285 (49.65) / 159 (49.66), 12-13	Quasi-experimental. 3-year assessment period (baseline (6th grade), 1-year (7th grade), 2 years (8th grade), 3 years (9th grade) follow-ups)	The entire class constitutes a team in arranged competitions with prizes. Different themes for each grade: Grade 4 (10- and 11-year-olds) traffic education; grade 5 (11- and 12-year-olds) morality and ethics and to prevent non-normative behavior such as delinquency, bullying, and shoplifting; and grade 6 (12- and 13-year-olds) encourages pupils to refrain from using alcohol, tobacco, and other drugs.	Focused on one of the three components of the Triad program – preventing alcohol use (“Fighting Drugs”). Include (1) increasing resistance skills through understanding and awareness of the harmful consequences of drugs and delinquency, (2) involving parents, and (3) a lottery contest where the whole class can win prizes.	Did not find an effect on the likelihood of drinking alcohol or drinking to intoxication.
	Primavera / Diaz et al. (2021). / France	School**	287 (48.0) / 266 (48.0), 10-12	Randomized Controlled Cluster Study. 18-months assessment period (baseline (T1), 6 months (T2), 18 months (T3) follow-up)	It runs over a three year period (final year of primary school to the second year of secondary school). Deployed over seven two-hour sessions with a facilitator trained in the program’s implementation. First three sessions in primary school, final session in secondary school.	It uses health promotion and is mainly based on experiential learning via the development of psychosocial skills (self-esteem, self-efficacy, motivation, adaptation strategies, and empowerment). The merger of several prevention programs: “GymSAT” (8–10 years old), “A vos marques, prêts, Santé!” (10–11 years old), “ICCAR” (12–13 years old).	Children were less likely to report current alcohol use at the end of the first year and past-month alcohol use at the end of the second year.
	Bridges/Puentes / Gonzales et al. (2018). / United States	School**	276 (51.2) / 144 (51.2), 17.9	Randomized controlled trial, three cohorts. 5-year assessment period (baseline	Integrates 3 components delivered in 9 weekly sessions at the adolescents’ schools: (1) an adolescent coping intervention, (2) parenting intervention, and (3) family strengthening intervention.	Grounded in ecodevelopmental systems and risk and protective factor frameworks and modeled on the format of the Strengthening Families Program, it emphasizes risk reduction (prevention) and positive youth	Significantly reduced the likelihood of an alcohol use disorder and reduced the frequency of alcohol use and drunkenness among youth who

			(T1), 5 years (T2) follow up)		development (promotion) in multiple domains (family, school, and peers).	reported any previous substance use.
Pragmatic prevention / <br>Hodder et al. (2017). / <br>Australia	School**	1261 (50.2) / 844 (49.7), 12-13	Cluster Randomized Controlled Trial. <br>3-year assessment period (baseline (T1), 3 years (T2) follow up)	Online survey. School staff should implement available programs and resources to target individual and environmental 'resilience' protective factors.	Sixteen broad strategies seeking to build the protective factors (individual or environmental) of students, implemented across the three domains of the Health Promoting Schools framework.	No difference in the prevalence of any measure of alcohol use between prevention and control students, nor was there any difference for aggregate or individual measures of individual and environmental protective factors.
Based on the Theory of Planned Behavior / <br>Kim et al. (2021). / <br>South Korea	School	170 (51.8) / 81 (45.7), 9	Quasi-experimental. <br>Transversal, pre-post	Web-based alcohol drinking prevention program. Five weekly school and home program sessions conducted over 5 weeks. The school program sessions lasted approximately 40 min each. The home program sessions lasted 20-30 min each.	The Theory of Planned Behavior (TPB) predicts intentions toward behavior simply and accurately through attitudes, subjective norms, and perceived behavioral control (PBC).	Significant improvements in attitudes and intention toward alcohol drinking prevention behavior.
Mantente REAL / <br>Kulis et al. (2020). / <br>Mexico	School**	735 (49.0) / 683 (49.0), 12	Cluster Randomized Controlled Trial. <br>Transversal, pre-post	Twelve weekly lessons that middle school teachers deliver in regular classes. A bilingual team trained 17 teachers to deliver the MREAL (two days) curriculum with fidelity.	Integrates ecological risk and resiliency theory, communication competence theory, and narrative theory to teach skills for resisting risky behaviors. <br>Delay or reduce substance use by increasing the use of culturally congruent drug resistance skills and promoting non-permissive substance use norms and attitudes.	Relatively less frequent use of alcohol. Higher risk students reported relative reductions in the frequency of alcohol use, especially males.
Preventure / <br>Lammers et al. (2017). /	School	343 (53.0) / 356 (43.0), 13-15	Cluster Randomized Controlled Trial.	Two 90-minute group sessions were carried out at the participants' schools. First group session: psycho-educational strategies were used to educate students about the target personality variable and	Selective prevention program with a personality-targeted approach. Brief intervention using motivational interviewing strategies and cognitive	Significant intervention effects were found to reduce alcohol use within the anxiety sensitivity

This is a provisional file, not the final typeset article

Netherlands			1-year assessment period (baseline (T1), 2 months (T2), 6 months (T3), 12-month (T4) follow-ups)	the associated problematic coping behaviors, such as risky behavior and substance misuse. Second session: participants were encouraged to identify and challenge personality-specific cognitive thoughts that lead to problematic behaviors.	behavioral skills training tailored to one of four personality profiles.	group and reduce binge drinking and binge drinking frequency within the sensation-seeking group.
Refuse, Remove, Reasons Program (RRR)<br><br>Mogro-Wilson et al. (2017). /<br><br>United States	School**	678 (48.0) / 674 (48.0), 15	Quasi-experimental.<br><br>Transversal, pre-post	Curriculum delivered by a trained facilitator over five weeks through five classroom sessions that addressed (1) alcohol and tobacco, (2) marijuana, (3) steroids and over-the-counter and prescription drugs, (4) heroin, and (5) consequences of use.	RRR uses social learning theory and the mutual aid model, a theoretical framework from the field of social work. RRR combines social resistance skills training and normative education.<br><br>RRR is the shorter prevention program of Mantente REAL.	Significantly reduced intoxication from alcohol, decreased social norms and acceptance of alcohol, and increased perceptions about negative consequences of alcohol use.
Fresh Start /<br><br>Onrust et al. (2017). /<br><br>Netherlands	School**	478 (55.4) / 548 (55.4), 12-13	Cluster randomized controlled trial.<br><br>9-month assessment period (baseline (T1), 5 months (T2), and 9 months (T3) follow up)	Four weekly lessons of 50 minutes each. First lesson: students discuss the transition to secondary school and Dutch legislation regarding substance use. Students receive a homework assignment to interview their parents about substance use. Second lesson: social norm discussion on substance use triggered by an interactive digital quiz. Third lesson: students practice decision-making skills by weighing the pros and cons of various decisions. Final lesson: erroneous assumptions regarding substance use are discussed.	Based on theories commonly used in health promotion: Health Belief Model, Theory of Planned Behavior, and Transtheoretical Model of Behavior Change. Focuses on attitudes toward substance use.	Small but significant effects on the attitudes toward alcohol.
Brief Intervention based on Motivational Interviewing (BIMI) /	School	116 (47.0) / 102 (47.0), 14	Randomized controlled trial.<br><br>6-month assessment period (baseline (T1), 3, months (T2), 6 months (T3) follow-up)	Each of the experimental group participants was attended to individually for an approximate time of 15 min: they were asked about personal experience in consumption and their motivation to change or maintain non-consumption. Psycho-education was provided. From there, the participant	Seeks to identify a present or latent problem about consumption and from there motivate the person to carry out a change.<br><br>Brief Interventions (BI). Motivational Interviewing (MI) is a client-centered and directive method approach that seeks to foster intrinsic motivation and	Significant reduction of risk levels of alcohol consumption 6 months later.

Reyes-Rodríguez et al. (2019). / Colombia				established a goal that had to do with decreasing the consumption of alcohol.	encourage change through ambivalence management and analysis.	
Unplugged / Sanchez et al. (2017). / Brazil	School**	3340 (51.3) / 3318 (51.3), 13	Randomized Controlled Trial. 9-months assessment period (baseline (T1), 9 months (T2) follow-up)	Implemented through 12 weekly 1-h lessons and is supported by school teachers (16-h training).	#Tamojunto. Based on a complex model (social influence model) that integrates theories such as social learning theory, problem behavior theory, the health belief model, the theory of reasoned action, and social norms theory, supporting the development of general social skills that are thought to reduce the effects of social influence by modifying attitudes, beliefs, and normative perception.	The program seemed to increase alcohol use initiation.
Unplugged / Sanchez et al. (2018). / Brazil	School**	2155 (51.3) / 2279 (51.3), 13	Randomized Controlled Trial. 21-month assessment period (baseline (T1), 21 months (T2) follow-up)	The same as Sanchez et al. (2017).	The same as Sanchez et al. (2017).	Increase in alcohol use in prevention and control groups 21 months later.
Unplugged / Sanchez et al. (2019). / Brazil	School**	3243 (51.3) / 3148 (51.3), 11-15	Randomized controlled trial. 21-month assessment period (baseline (T1), 9, months (T2), 21 months (T3) follow-up)	The same as Sanchez et al. (2017).	The same as Sanchez et al. (2017).	The program's effect itself on alcohol use via normative beliefs was not found to be statistically significant.
Unplugged / Valente et al. (2019). /	School**	3340 (51.3) / 3318 (51.3), 11-15	Randomized controlled trial. 21-month assessment period (baseline (T1), 21	The same as Sanchez et al. (2017).	The same as Sanchez et al. (2017).	The intervention effects are implausible to be conditioned to either

This is a provisional file, not the final typeset article

Brazil			months (T2) follow up)			parenting style dimension.
The GOOD Life / Vallentin-Holbech et al. (2019). / Denmark	School	641 (53.0) / 714 (54.9), 13-17	Cluster randomized controlled trial. 3-months assessment period (baseline (T1), 3 months (T2) follow-up)	Social norms messages through three communication elements: classroom feedback sessions, posters, and web application.	Based on the social norms approach. Assess pupils’ perception of peer lifetime binge drinking, frequency of binge drinking, and alcohol-related harms.	The prevention effect was insignificant for the frequency of binge drinking. For binge drinking, no significant effects for any level of exposure, satisfaction, or recall were found. Higher levels of satisfaction and exposure and the female sex were associated with better recall of the intervention.
Unplugged / Vigna-Taglianti et al. (2021). / Nigeria	School**	1121 (44.8) / 1564 (34.0), 14.2	Cluster randomized controlled trial. Transversal, pre-post	The same as Sanchez et al. (2017).	The same as Sanchez et al. (2017).	Significantly reduced the prevalence of recent alcohol use. The program prevented progress and encouraged regress across stages of the intensity of alcohol use. Negative beliefs, risk perceptions, and class climate mediated the effect of Unplugged on alcohol use.
Media Detective Family / Scull et al. (2017). /	Family**	47 (52.0) / 36 (52.0), 9.9	Randomized Controlled Trial. Pre-post and three-month follow-up.	It is built around solving a problem with a plot and story that ties the program's parts together. Parents and their children are trained together to become media detectives who can solve a series of mysteries related to advertising.	Designed to strengthen children’s logical responses to media messages and make youth more aware of their emotional reactions to the media, hypothesize that beliefs and expectancies about risk behaviors shape	Children reported a significant reduction in their use of alcohol over time.

United States					and motivate both the intent to use and the actual use of substances.	
Effekt / Tael-Öeren et al. (2019). / Estonia	Family	479 (50.6) / 503 (51.5), 11-13	Cluster randomized controlled trial. 3-year assessment period (baseline (T1), 18-months (T2), 30-months (T3) follow-ups)	Six meetings, two per year, were held at all schools by qualified trainers, who underwent intensive training throughout the program.	To delay and reduce adolescents' alcohol use by maintaining parental restrictive attitudes towards adolescents' alcohol use over time.	Positive effect on parental attitudes, but it did not delay or reduce adolescents' alcohol consumption.
Based on the Theory of Planned Behavior / Park et al. (2021). / South Korea	Communit y	Girls, 23/22, <19	Quasi-experimental. Transversal, pre-post	Six sessions, with one-hour sessions twice a week, involved group lectures and small group activities.	Behavioral intention is the direct determinant of switching to healthy behavior, and individuals with solid intentions make efforts to achieve a goal. They are more easily motivated to change their behavior. Behavioral intention is influenced by an attitude based on beliefs about behavior, social norms, and perceived behavioral control.	Improved alcohol-related knowledge and converted individuals' positive expectations of alcohol to negative ones.
Öckerö Method / Svensson et al. (2021). / Sweden	Communit y	2016: 519 (50.1) /534 (48.1), 7-9 grades 2017: 474 (50.1) / 497 (48.1), 7-9 grades 2018: 487 (50.1) / 527 (48.1), 7-9 grades	Quasi-experimental. 3-year assessment period (baseline (T1), 1 year (T2), 2 years (T3), 3 years (T4) follow-up)	Implemented by local prevention workers, followed up using self-report surveys conducted once each year with adolescents in secondary school.	To change social norms of adolescents with regards to alcohol consumption by providing information to parents, other adults, local associations, and local media to influence adolescents' attitudes towards alcohol consumption.	No effect on adolescents' drinking habits or their perceptions of their parents' attitudes towards adolescent drinking.
RealTeen / Schwinn et al. (2017). /	Web-based**	Girls, 394/394, 13-14	Randomized controlled trial. 1-year assessment period (baseline	Nine intervention sessions. Two components: the homepage and the intervention sessions. The homepage for the program was accessible at any time, including feeds from the latest entertainment sites, online polls,	Based on Social Learning Theory. Focused on goal setting, decision making, puberty, body image, coping, drug knowledge, refusal skills (two sessions), and a review.	Girls reported less binge drinking and higher alcohol skills, coping skills, and media literacy.

This is a provisional file, not the final typeset article

United States			(T1), 1 year (T2) follow-up)	horoscopes, beauty tips, and a quote of the day.		
RealTeen / <br>Schwinn et al. (2019). / <br>United States	Web-based**	Girls, 394/394, 13-14	Randomized controlled trial. <br>3-year assessment period (baseline (T1), 1 year (T2), 2 years (T3), 3 years (T4) follow-up)	Nine intervention sessions. Two components: the homepage and the intervention sessions. The homepage for the program was accessible at any time, including feeds from the latest entertainment sites, online polls, horoscopes, beauty tips, and a quote of the day.	Focused on goal setting, decision making, puberty, body image, coping, drug knowledge, refusal skills (two sessions), and a review.	Reductions in binge drinking occurred at 1-year follow-up.

** At baseline; ** Program applied to other drugs besides alcohol.*

1
